# Development of Landmark Use for Navigation in Children: Effects of Age, Sex, Working Memory and Landmark Type

**DOI:** 10.3390/brainsci12060776

**Published:** 2022-06-13

**Authors:** Anne H. van Hoogmoed, Joost Wegman, Danielle van den Brink, Gabriele Janzen

**Affiliations:** 1 Behavioural Science Institute, Radboud University Nijmegen, 6525 XZ Nijmegen, The Netherlands; joostwegman@gmail.com (J.W.); dannie.vandenbrink@radboudumc.nl (D.v.d.B.); 2 Donders Institute for Brain, Cognition, and Behaviour, Radboud University Nijmegen, 6525 XZ Nijmegen, The Netherlands

**Keywords:** spatial navigation, virtual reality, individual differences, spatial cues, development, working memory

## Abstract

The use of landmarks for navigation develops throughout childhood. Here, we examined the developmental trajectory of egocentric and allocentric navigation based on landmark information in an on-screen virtual environment in 39 5–6-year-olds, 43 7–8-year-olds, and 41 9–10-year-olds. We assessed both categorical performance, indicating the notion of location changes based on the landmarks, as well as metrical performance relating to the precision of the representation of the environment. We investigated whether age, sex, spatial working memory, verbal working memory, and verbal production of left and right contributed to the development of navigation skills. In egocentric navigation, Categorical performance was already above chance at 5 years of age and was positively related to visuo-spatial working memory and the production of left/right, whereas metrical performance was only related to age. Allocentric navigation started to develop between 5 and 8 years of age and was related to sex, with boys outperforming girls. Both boys and girls seemed to rely more on directional landmark information as compared to positional landmark information. To our knowledge, this study is the first to give insight into the relative contribution of different cognitive abilities to navigation skills in school-aged children.

## 1. Introduction

Successful wayfinding is a crucial skill for everyday life. Both humans and animals have several mechanisms at their disposal to support successful navigation (e.g., [1]). In the current study, we examined the development of these mechanisms and their cognitive correlates in primary school children. In the literature on spatial cognition, multiple terms are used to describe navigation mechanisms. Based on studies on animal spatial learning, Wang and Spelke [2] suggest that human navigation depends on three processes: path integration, view-dependent place recognition, and geometry-based reorientation. They describe path integration as ‘a process by which the relation of the animal to one or more significant places in the environment is updated continuously as the animal moves’ (p. 376). View-dependent place recognition is described as the use of snapshot view-matching, while reorientation is described as the use of the shape of an environment to restore the spatial relationship between an individual and its environment. Path integration and view-dependent scene recognition both rely on the relation between the navigator and the environment, and can thus be described as egocentric processing. Wang and Spelke [2] propose that geometry-based reorientation is based on the shape of the environment only, and can thus be described as allocentric processing. However, allocentric processing does not necessarily depend on geometry only, as it can also be based on (distal) landmarks within the environment [1]. In this study, we will use the term egocentric navigation to refer to navigation based on path integration (i.e., updating ones location based on self-motion cues) and viewpoint matching. We will use the term allocentric navigation to refer to navigation based on the use of external cues in the environment to establish a map-like representation. It is important to note that landmarks can be used for both egocentric and allocentric navigation.

Previous studies in adults found evidence for navigation skills to be related to sex, spatial working memory, verbal working memory, and language skills. While many studies show an effect of sex, with men outperforming women on allocentric navigation tasks [3,4,5,6]; for a review see [7,8,9], the relations between navigation skills and spatial working memory, verbal working memory, and language skills are inconclusive [10,11,12,13,14]. In children, associations are even less clear. While some studies show sex differences in navigation skills favoring girls, others show no such difference in performance [15,16]. Further studies observed sex differences favoring boys [17]. A recent study used a virtual spatial navigation task and observed that boys were more accurate and navigated faster than girls [18]. In line with findings by Newcombe [9], this difference between girls and boys between 9 and 11 years increased with age. Furthermore, some research indicates that language skills are related to certain forms of navigation [19,20], although other studies suggest that this is not the case [21,22]. To our knowledge, spatial working memory and verbal working memory have not been previously associated with navigation skills in children. However, during navigation, one needs to keep track of ones orientation within space, either by using verbal updating or using a spatial updating. Therefore, in the current study, we examined individual differences in egocentric and allocentric navigation skills in children by relating them to age, sex, verbal working memory, visuo-spatial working memory and language skills. 

In the 1960s, Piaget and Inhelder studied the development of spatial skills and proposed that egocentric processing was innate, whereas allocentric processing was acquired later in life [23]. Since then, a large and growing body of literature has investigated navigation skills in children (see [24] for a review), mainly focusing on landmark use for egocentric and allocentric navigation. Egocentric navigation based on landmarks starts with beacon use (i.e., landmarks that directly mark the goal location) already before the age of one year [25,26,27]. Moreover, the use of the geometry of the environment to reorient (whether or not in combination with landmarks) has been shown to start early in life, and to develop between 1.5 and 6 years of age, with the joint use of landmarks and geometry depending on the size of the environment and distance between the landmark and the child [21,22,28,29,30,31,32,33]. Landmark use in environments in which geometry information is not helpful has been studied less extensively but seems to emerge between 8 months and 3 years of age. However, the age at which landmark use can be shown depends on the difficulty of the paradigm used [33,34,35,36,37]. While the use of landmarks in the above-mentioned studies is often interpreted as evidence for allocentric processing, Nardini, Thomas, Knowland, Braddick and Atkinson [38] suggest that, in most of these cases, egocentric processing would also allow for finding the goal location. In an attempt to investigate true allocentric processing, in their study, a large asymmetric landmark was placed in the middle of a room with one box placed on either side serving as hiding locations. Children hid the object in one of the two boxes after which they were disoriented (preventing path integration) and with their eyes closed were either moved back to the original location from where they hid the object or were moved to the exact opposite side of the room facing the ‘back’ of the landmark. In the condition in which they were moved to the opposite side, viewpoint-matching was not possible. Having to rely on viewpoint-independent processing when faced with the alternative side of the landmark, 6- to 8-year-olds were able to find the goal location. However, a younger age group of 4- and 5-year-olds was not. Although Nardini et al. [38] point out that viewpoint-independent processing in this study is possibly not based on a mental map but is more limited in scope, the results suggest that true allocentric processing only starts to develop between 5 and 6 years of age. In a recent study, Negen, Heywood-Everett, Roome, and Nardini [39] used an immersive virtual reality task to avoid additional task demands of a real environment and found that allocentric recall was possible only for their older age group (4.0–4.5) but not in younger children between 3.5 and 4 years of age (for a review see [40]). In the current study, we further examined the development of allocentric processing in school-aged children. Whereas Nardini et al. [38] used fixed visual hiding positions (boxes), in the current study, we used a virtual open environment without distinguishable target locations, similar to Negen and colleagues [39] (but also in other studies with adults [41,42]), which enabled us to examine individual differences in the precision of egocentric and allocentric navigation in children. 

Allocentric representations based on landmarks contain different types of information, i.e., positional and directional information. Positional cues are unique landmarks that are close to the navigator and, therefore, provide detailed information about the location of the navigator within the environment. Directional cues mainly provide information about the direction one is heading in [43]. Examples of the latter type of cues are distal landmarks, slants, and shadows. In adults, research has shown that men prefer to base their navigation on directional landmarks, whereas women prefer to rely on positional landmarks [4,44,45,46] (see [47] for neural correlates of landmark use across development). In the current study, we investigated children’s use of positional and directional cues by modulating their presence.

We examined sex differences in navigation, as well as the relation between navigation skills, working memory and language skills. In adults, concurrent spatial working memory tasks have been shown to impede navigational performance, whereas influences of concurrent verbal working memory tasks and verbal shadowing tasks on navigation are mixed [10,11,12,13,14]. In children, no association has been found between verbal short-term memory and navigational performance [20], but associations with verbal working memory have not directly been investigated. With regards to language skills, the number of spatial prepositions 16- to 24-month-old children know (e.g., behind) was found to be related to landmark use in a paradigm based on the Morris Water Maze [19]. Moreover, in 5.5- to 6.5-year-old children, the correct verbal production of the terms left and right, but not comprehension of the concepts left and right has been found to be related to landmark use in a reorientation task [20]. These studies suggest that certain language skills are associated with navigational ability in children. However, while the use of landmark information in reorientation previously also has been believed to build on the development of language [11], more recent studies revealed that even very young children who are not able to use left and right as well as monkeys were able to use landmark information [31,36,48]. 

**Figure 1:**
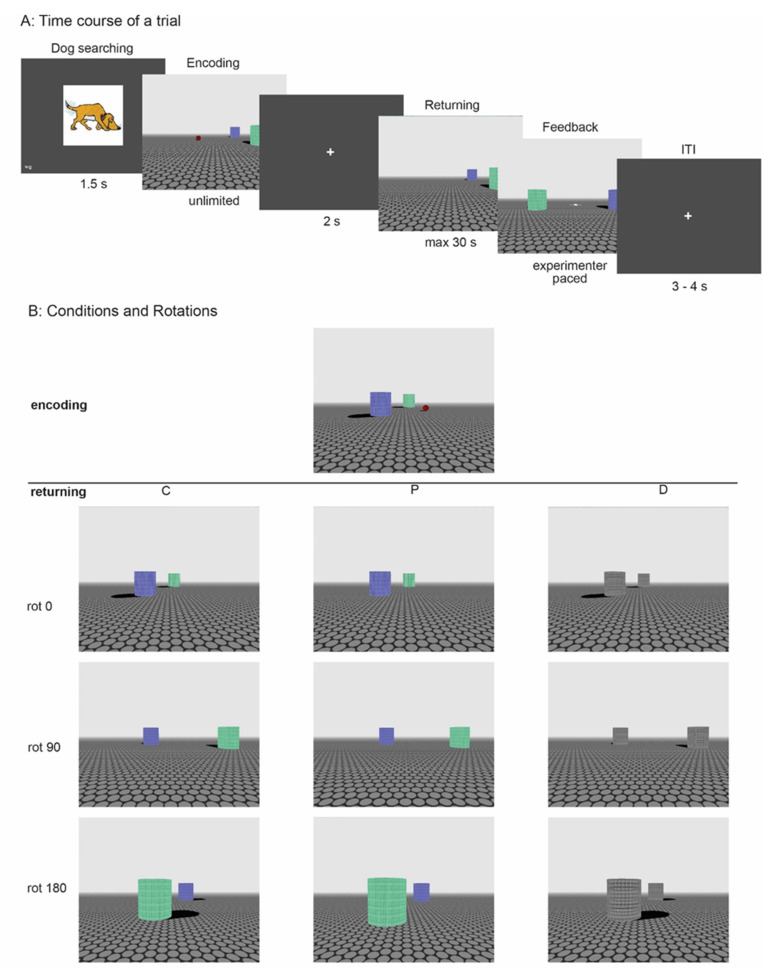

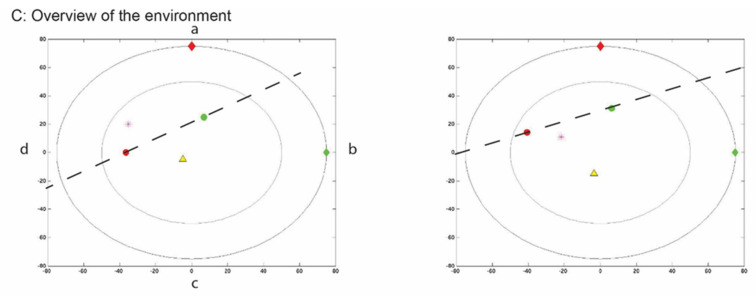
The navigation task: (**A**) Time course of a trial; (**B**) snapshots of the starting position for crossed cue types (combined, positional, and directional) with rotation between encoding and returning phases (0, 90, and 180 degrees); (**C**) Two trials from one participant depicted from a birds eye perspective. The red and green dots represent the columns. The yellow triangle represents the location of the ball in the encoding phase. The green diamond represents the starting location in the encoding phase, and the red diamond represents the starting location in the returning phase. The purple star represents the location where the ball was placed in the returning phase. The circular lines were not visible in the environment. Letters **a**–**d** represent possible starting locations during encoding and returning.

In the present study, we examined the developmental trajectory of egocentric and allocentric navigation based on landmark information and investigated the aforementioned candidate cognitive factors contributing to this development in school-aged 5- to 10-year-old children. Based on a paradigm developed by Baumann et al. [41], we employed an on-screen virtual navigation task in an open environment with one green and one blue cylindrical landmark casting shadows on the floor (see Figure 1A). Trials consisted of an encoding phase in which a ball had to be located and retrieved by virtually moving through the open environment and a returning phase in which the child had to navigate to the original position where the ball previously was located in order to place it back. The starting point in the returning phase was either identical to the encoding phase or starting from a different viewpoint. Starting from an identical viewpoint enabled navigation based on an egocentric representation of the relation between the ball and the child. Starting from a different viewpoint required the use of an allocentric representation of the environment including the ball, the landmarks and the relations between these objects. Landmark information was manipulated during the returning phase such that either the directional or positional information of the columns was missing in certain trials (see Figure 1B). Data was coded both categorically and metrically to gain insight into whether the children were aware of the rotation/viewpoint manipulation, and to gain insight into the representational precision. Previous research suggests that categorical and metrical results may differ due to the involvement of different brain regions in categorical versus metrical location coding [49]. This is in line with an fMRI study by Baumann, Chan and Mattingley [50] who found the parietal cortex involved in the encoding of categorical locations and the medial temporal lobe as well as the striatum engaged in coordinate location memory.

After the navigation task, children performed a verbal working memory task, a spatial working memory task, and a left/right production task. Inclusion of these additional measurements allowed for a more comprehensive investigation of factors associated with the development of egocentric and allocentric navigation skills.

Our main hypothesis was that spatial navigation based on landmark information would increase with age in 5- to 10-year-olds. More specifically, we hypothesized egocentric navigation to be present by 5 years of age, and allocentric navigation to develop later, with the precision of allocentric representations to increase with age [26,51,52]. In addition, we explored the relative contributions of sex, verbal working memory, spatial working memory, and language skills to children’s egocentric and allocentric navigational abilities.

## 2. Materials and Methods

### 2.1. Participants

The sample consisted of 122 children across three age groups: 39 5-to 6-year-olds (20 boys, 19 girls) with a mean age of 5 years, 11 months (sd 5 months), 43 7-to 8-year-olds (22 boys, 21 girls) with a mean age of 8 years, 1 month (sd 6 months), and 41 9- to 10-year-olds (20 boys, 21 girls) with a mean age of 10 years, 1 month (sd 6 months). A power-analysis for the main analyses with a medium effect size (V = 0.25; other parameters α = 0.05 β = 0.8, number of groups = 6, number of measurements = 3) in G*power revealed a sample size estimation of 135 participants. Due to restraints on resources, we fell 13 participants short to meet the intended power.

Children were predominantly recruited from regular primary schools in the Netherlands. In addition, a small number of children in the youngest age group were recruited via the Baby and Child Research Center at the Radboud University Nijmegen. Written informed consent was obtained from the legal guardians for each child according to a protocol approved by the Radboud University Nijmegen Ethics Committee for Behavioural Research (ECG).

### 2.2. Measures

#### 2.2.1. Navigation Task

The navigation task was an adapted version of an on-screen virtual navigation task developed by Baumann et al. (2010). A 3D environment was created with two differently colored columns as landmarks, and a ball placed on the floor, at a location close to the landmarks (see Figure 1A). Trials consisted of an encoding phase and a returning phase. In the encoding phase, a child was required to gather the ball by virtually moving through the environment to the location of the ball using the arrow keys on a keyboard to simulate retrieving the ball. Arriving at the exact location of the ball was rewarded with a sound and followed by a blank screen with a fixation cross for 2 s. In the returning phase, children were asked to place the ball at the exact same location they had previously picked it up by virtually moving to the remembered location and pressing the space bar to release the ball. A time limit was set to stimulate using the shortest route (i.e., preventing the child from using viewpoint matching processing by first moving towards the (invisible) original starting location in the encoding phase). If the child did not place the ball back in the virtual environment within 20 s, an orange square appeared in the top right corner of the screen to indicate that the child was almost out of time. If the ball was not placed within an additional 10 s, the square would turn red, a sound was played, and the trial was ended. Upon timely placement of the ball, a rewarding sound was played, and the child was encouraged to use the left and right arrow keys to search for the white cross on the floor, which revealed feedback on the location the ball should have been placed (see Figure 1A). 

Whenever the ball was placed at or very close to its original location, a picture of a happy dog was shown. The feedback phase was terminated by the experimenter when he or she observed that the child had seen the target location. A fixation cross appeared marking the inter-trial interval of 3 to 4 s before the next trial started. 

The experiment consisted of 32 trials across three conditions that were intermixed; trials containing combined (C) cues, positional (P) cues only, and directional (D) cues only (see Figure 1B). In the encoding phase, both positional (i.e., colored columns) and directional cues (shadows) were visible. During the returning phase, in the C condition, both cues remained present. In the P condition, the color remained, but the shadows were absent. In the D condition, shadows remained, but now color information was lacking. Sixteen trials were presented in the C condition which functioned as a baseline condition, whereas eight trials were presented in the P and D conditions. Next to best resembling a natural environment, the C condition was easier for the children, which enhanced their willingness to finish the task.

Next to manipulating landmark availability, the participants’ starting position between the encoding and returning phase was also manipulated. In 8 trials, participants started from the same location as they had started from during encoding, in 8 trials they entered the environment after a 90-degree clockwise rotation around the environment, in 8 trials they entered after a 180-degree clockwise rotation, and in 8 trials they entered after a 270-degree clockwise rotation. Whereas starting from the same location enabled the use of egocentric navigation, after rotation, allocentric navigation was necessary to find the goal location. The rotations were distributed equally across cue conditions (see Figure 1B). In addition, within the experiment, distance to the target in the returning phase, position of the target either in front of or behind the landmarks during encoding, and distance of the target to the landmarks were counterbalanced.

##### Setup

The task was constructed in an open-source 3D and animation suite Blender (The Blender Foundation, Amsterdam, The Netherlands). All sizes and distances are measured in Blender units. The virtual environment in which the task was executed consisted of an infinite area with a gray-black spotted floor. Above the floor, the environment was covered with fog around the participant starting at 100 units away from the participant and being completely dense at 200 units away. Two landmarks were present in the environment, a blue column and a green column. The columns were 11 units wide and 12 units high. They were both casting shadows on the floor. The location of the columns was different in every trial, but the columns were always 50 units apart from each other and both placed within the inner circle of two invisible circles (with a diameter of 100 and 150 units) in the environment which were used for the generation of the trials (see Figure 1C). A ball with a diameter of 5 units was placed 20 or 40 units away from and perpendicular to the middle of an imaginary line between the two landmarks and was also within the inner invisible circle. The starting point of the participants was on the outer circle of the environment, facing inwards, always at a distance of 80 units away from the target during encoding. In the returning phase, the distance to the ball was counterbalanced between 80 and 90 units.

##### Instructions

Before the task started, the children were first familiarized with the arrow keys on the keyboard and the task. At the start of the task, the child was told that a dog was searching for a ball and that it needed some help. The child could help by getting the ball for the dog. The trial then started with a picture of the dog and a sound of a sniffing dog for 1.5 s after which the encoding phase started. The child was told to attend to the columns and their shadows to remember where the ball was placed. The child was instructed to pick up the ball as quickly as possible. Following the encoding phase, the child was told to return the ball to the same place where it was picked up because the dog wanted to retrieve the ball itself afterwards. Then the returning phase started.

During the familiarization phase, the feedback system was introduced to the child. The researcher explained to the child that after placing the ball, a cross on the floor indicated the location where the ball should have been placed. Moreover, he or she would see a happy dog accompanied by a happy sound after the trial when they placed the ball at the right location (which was within 10 units from the target location). Whenever the happy dog appeared, the child was rewarded offline. He or she was instructed to place a marble in the dog’s kennel, which was made of cardboard. After the experiment, the researcher and the child would count the number of marbles gained during the experiment. The child was verbally rewarded and encouraged throughout the task.

#### 2.2.2. Working Memory Task

Two subtests of a Dutch translation of the Automated Working Memory Assessment (AWMA; [53]) were administered. The internal validity of the AWMA is sufficient for children between 4 and 11 years of age. First, the ‘backwards digit recall’ subtest was administered to measure verbal working memory. The children were instructed by the computer to recall the digits in the opposite order in which they had been presented. After the practice trials, a block with 2-digit strings was presented. A maximum of six trials was presented within a block. After four correct answers, the next block started, containing series with one extra digit. After 3 wrong answers within a block, the task was terminated.

To measure visuo-spatial working memory, the ‘odd-one-out’ subtest was administered. In this task, children saw a grid with 3 pictures, two of which were identical. They had to indicate which picture was the odd one out. After that, an empty grid was shown on which children were required to indicate where the deviating picture had been presented before. In the next block, the first two grids of 3 pictures were presented after each other, and only after they had indicated both odd ones, an empty grid was shown. In this empty grid, they had to indicate where the odd ones had been presented in the same order as they were presented. The blocks of trials were presented via the same procedure as in the backwards digit recall task. For both tasks, the experimenter checked with the manual whether a solution was correct or incorrect and entered this into the computer. After the test, a score was computed automatically. The standardized scores with a mean of 100 and an sd of 15 in the population were used in the analyses.

#### 2.2.3. Left-Right Task

The left-right task was based on the left-right task used by Hermer-Vaszquez, Moffet, and Munkholm [20]. On a light blue background, one multi-colored ball was presented in the middle of the screen. In addition, in each trial, a single-colored ball was presented either above, below, left or right from the multi-colored ball. The pictures were made in Blender and presented with Powerpoint (Microsoft Office). The task consisted of 16 trials and one practice trial. On each trial, the children were asked: ‘Can you tell me where the single-colored (e.g., black) ball is with respect to the multi-colored ball in the middle?’ If the child answered that the single-colored ball was ‘next to’ the multi-colored ball, the experimenter would ask: ‘Can you also tell me which side?’ Almost all children then indicated left or right, and these answers were used to compute the scores. Scores were added for the left-right items leading to a score ranging from 0 to 8. Afterwards, the scores were dichotomized. A score of 8 was transformed into a score of 1 while a score lower than 8 was transformed into a score of 0. The above-below items were only used as fillers.

#### 2.2.4. Parental Questionnaire

The parents of all participating children were asked to fill out a questionnaire. The questionnaire asked the parents for some background information about the family as well as the navigation skills of their child, how their child typically went to school, and the computer skills of their child. With regard to computer skills, parents indicated on a five-point Likert scale how proficient their child was in using a keyboard. Moreover, they were asked for the number of hours per week their child played computer games.

### 2.3. Procedure

Participants were mainly recruited via regular primary schools in the eastern part of the Netherlands. Additional participants were recruited through the Baby and Child Research Centre of Radboud University. Parents signed a consent form if they allowed their child to participate. The children were tested individually on a laptop in a separate room at their school (or in a few cases at the university). First, the navigation task was administered. This took about 25 min. Next, the working memory tasks and the left-right task were administered, always in the same order. The total testing session lasted approximately 45 min. The children were rewarded with a small toy for participating. 

### 2.4. Data Analyses

Data were analyzed according to metrical (precision) and categorical errors. Metrical errors were measured as the distance between the target location during encoding and the location at which the child placed the ball in the returning phase. For the categorical analyses, we observed whether the ball was placed on the same side of the landmarks as the target location. In order to do so, an imaginary line was drawn through both landmarks (see Figure 1C). If both locations were on the same side of this line, the trail was scored as correct, if not, an error was scored. In each condition, the percentage of correct answers was computed. This analysis was added to gain more insight into the type of errors that were made. If one would use the landmarks when returning the ball, it should be at the correct side of the landmarks regardless of the distance to the correct location. If not, the ball could be placed equally far from the correct location but is more likely to end up on the other side of the landmarks.

For the rotations, the 90- and 270-degree turns were taken together and labeled as 90-degree turns. Data were analyzed separately for Rotation and Cue Type to avoid unreliable means due to low numbers of trails per cell. 

Two repeated measures MANOVAs with both metrical and categorical error as dependent variables were carried out with Rotation (0, 90, 180 degrees) or Cue Type (C, D, P) as within-subject factor and Age Group and Sex as between-subject factors to assess the development of egocentric versus allocentric navigation and preferred cue type, respectively. Greenhouse–Geisser correction for nonsphericity was applied whenever appropriate. Corrected *p* values are reported along with original degrees of freedom. Post-hoc analyses for Age Group were Sidak-corrected [54]. Planned contrasts were used to analyze the difference between 0- and 90-degree rotations and 90- and 180-degree rotations, as well as between the combined cues and positional cues (C vs. P), and combined cues versus directional cues (C vs. D). With regard to the Rotations, we were also interested in the relationship between performance in different rotations and verbal working memory, spatial working memory, and left/right knowledge. In addition, we checked whether the performance was related to the ability to use a keyboard and the hours of game playing of the children as reported by the parents. Therefore, we performed two MANCOVAs, one on metrical performance and one on categorical performance, with Rotation as a within-subjects factor, and Age (in days), Sex, verbal working memory, spatial working memory, left/right knowledge, keyboard use (dichotomized), and hours of gaming as covariates.

## 3. Results

Ten 5- to 6-year-olds (4 boys, 6 girls) were excluded from the analyses because they were unable to finish the navigation task. These children did not differ significantly from their included peers in verbal and spatial working memory or left-right knowledge. Results are presented separately for rotations and cue type. Descriptive statistics on the additional behavioral tasks are reported in Table 1.

### 3.1. Rotations

**Figure 2:**
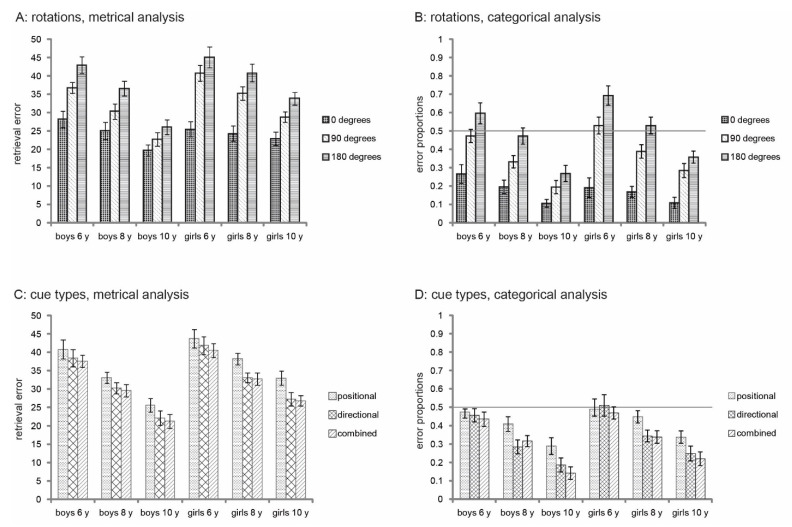
Results for all Age *Sex groups on the cue types and rotations displaying means and standard errors. (**A**,**C**) present the retrieval errors in Blender units. (**B**,**D**) present the categorical errors in proportions. The horizontal line in (**B**,**D**) represents the chance level with bars higher than this level representing at/lower than chance level.

The results for the 0, 90, and 180-degree rotations are presented in Figure 2A and 2B. The results of the repeated measures MANOVA with Rotation as a repeated measure, Age and Sex as between-subjects measure and categorical and metrical error as dependent variables are reported in Table 2. 

The MANOVA revealed an effect of Rotation, an effect of Sex, and an effect of Age Group. Moreover, an interaction between Rotation and Sex and a marginal interaction between Rotation and Age Group were found. Univariate tests showed that all main effects applied to both metrical and categorical error data, except for Sex, which was only marginally significant for the categorical data. Planned contrasts showed a difference between both 0- and 90-degrees rotation, both *Fs* > 48.66, *p* < 0.001, *η*^2^ > 0.314, and 90- and 180-degrees rotation, both *Fs* > 25.22, *p* < 0.001, *η*^2^ > 0.192. Post-hoc analyses showed that the 5- to 6-year-olds differed from the 7- to 8-year-olds, and the 7- to 8-year-olds differed from the 9- to 10-year-olds on both the metrical errors, both ps < 0.001, and categorical errors, *p* = 0.001, and *p* < 0.001, respectively. Planned contrasts on the interaction between Rotation and Sex showed a difference between boys and girls in the 0- vs. 90-degree rotations, metrical: *F* = 5.60, *p* = 0.020, *η*^2^ = 0.050, categorical: *F* = 7.16, *p* = 0.009, *η*^2^ = 0.063, but not in the 90- vs. 180-degree rotations, both metrical and categorical *F* < 1, n.s. For the metrical errors, girls and boys did not differ on the 0-degree rotation, t (110) = −0.066, *p* = 0.948, but boys made less errors on the 90-degree rotation, t (110) = −2.87, *p* = 0.005, r^2^ = 0.06. For the categorical errors, no significant difference between boys and girls on either the 0-degree rotation or 90-degree rotation was observed, t (111) = −1.08, *p* = 0.284, and t (111) = 1.64, *p* = 0.104, respectively, but the difference between the rotations was larger for girls as compared to boys. Planned contrasts on the interaction between Rotation and Age Group also revealed a difference between Age Groups in the 0-degree rotation versus 90-degree rotation, metrical: *F* (2, 105) = 3.24, *p* = 0.043, categorical: *F* (2, 105) = 3.48, *p* = 0.034, *η*^2^ = 0.058, but not in the 90- vs. 180-degree rotation, both *Fs* (2, 105) < 1.3, ps > 0.283. The difference between errors in the 0- and 90-degrees rotations decreased with age due to stronger improvement on the 90-degree rotation than on the 0-degree rotation.

Results from the MANCOVA on the metrical errors with the predictors Sex, Age, Verbal WM, Spatial WM, Left/right knowledge, Keyboard use, and Gaming experience showed an effect of age, and marginal effects of sex and spatial working memory on the precision of navigational performance (see Table 3). More specifically, age was positively related to performance in the 90- and 180-degree rotations, both *Fs* (1, 87) > 17, *p* < 0.001, *η*^2^ > 0.167 but was only marginally significant for errors in the 0-degree rotation, *F* (1, 87) = 2.86, *p* = 0.095, *η*^2^ = 0.032. Sex was not related to performance in the 0-degree rotation, *F* (1, 87) = 0.55, *p* = 0.460, marginally significant in the 90-degrees rotation, *F* (1,87) = 3.75, *p* = 0.056, *η*^2^ = 0.041, and significant in the 180-degrees rotation, *F* (1, 87) = 6.53, *p* = 0.012, *η*^2^ = 0.070, indicating better performance in boys as compared to girls. Spatial working memory was only positively related to performance in the 0-degree rotation, *F* (1, 87) = 5.73, *p* = 0.019, *η*^2^ = 0.062, but not in the 90- and 180-degree rotations, both *Fs* (1, 87) < 1, n.s.

For the categorical errors, results of the MANCOVA showed significant effects of age, spatial working memory, and left/right knowledge (see Table 3). Age was positively related to performance in the 90- and 180-degree rotations, both *Fs* (1, 88) > 13, *p* < 0.001, *η*^2^ > 0.129, but not in the 0 degrees rotation, *F* (1, 88) = 2.64, *p* = 0.108. Spatial working memory was only positively related to performance in the 0 degrees rotation, *F* (1, 88) = 7.68, *p* = 0.007, *η^2^* = 0.080, but not in the 90- and 180-degree rotations, both *Fs* (1, 88) < 1, n.s. Left/right knowledge was positively related to performance in the 0-degrees rotation, *F* (1, 88) = 6.67, *p* = 0.011, *η*^2^ = 0.070, and in the 180 degrees rotation, *F* (1, 88) = 9.12, *p* = 0.003, *η*^2^ = 0.090, but not in the 90 degrees rotation, *F* (1, 88) = 1.96, *p* = 0.165.

### 3.2. Cue Type

Errors in the positional (P), directional (D), and combined (C) conditions are presented in Figure 2C,D for each age group and sex separately.

Statistical results of the repeated measures MANOVA with Cue Type as a repeated measure, Sex and Age-group as between-subject measures and metrical and categorical errors as dependent variables are reported in Table 4. An effect of Cue Type was revealed for both metrical and categorical errors. Planned contrasts revealed a difference between the C and P condition, both *Fs* (1, 106) > 18.98, *p* < 0.001, *η*^2^ > 0.152, but not between the C and D condition, both *Fs* (1, 106) < 1, n.s., with larger errors in the P condition compared to the D and C conditions. Moreover, significant main effects of Sex and Age Group indicated that boys made smaller/less errors than girls, and that task performance improved with age. Post-hoc comparisons showed that 9- to 10-year-olds outperformed both 5- to 6-year-olds and 7- to 8-year-olds, all ps < 0.001, and that 7- to 8-year-olds outperformed 5- to 6-year-olds for metrical, *p* < 0.001, and categorical *p* = 0.001 performance. No significant interactions were found.

## 4. Discussion

In the current study, we investigated the development of allocentric and egocentric navigation based on landmarks in 5- to 10-year-olds, as well as the relation with individual-level factors age, sex, verbal working memory, spatial working memory, and left/right knowledge to these skills. 

Both the metrical and categorical results showed an increase in performance with age, indicating that, in general, children become better at navigating based on proximal landmarks as they mature. This indicates that their mental map becomes more precise with age. Navigational performance was better for all age groups when starting from the same starting point (0-degree rotation), as compared to starting from a different starting point (90- or 180-degree rotation), indicating that children, in general, do better in this task when egocentric navigation based on viewpoint matching and path integration is possible in addition to allocentric navigation. Performance was better after 90-degrees rotation as compared to 180-degrees rotation, possibly due to the easier detection of a change in location and/or less difficulty in spatial updating of the children’s egocentric representation after starting from a 90-degree rotation relative to the 180-degree rotation. Detection of the 180-degree rotation may have been difficult, especially for the younger children. However, the difference in performance between 0- and 90-degrees rotation, in which detection of the change was much easier, suggests that this cannot fully explain the difference in egocentric versus allocentric navigation. A preference for egocentric navigation is in line with findings in adults navigating through unfamiliar environments (e.g., [55]).

More importantly, a difference in developmental trajectories of egocentric versus allocentric navigation was found. Allocentric navigation was shown to develop at a later age as compared to egocentric navigation in this paradigm, with 9-to-10-year-olds showing a smaller difference between egocentric and allocentric than 5-to-8-year-olds. Egocentric navigation was already present in 5-to-6-year-olds, but performance still improved with age, whereas allocentric navigation seemed to be at a chance level in the 5-to-6-year-olds. In line with other studies, these results suggest that egocentric processing develops earlier than allocentric processing [39,51,52]. Our results showing that allocentric navigation based on proximal landmarks in an open environment starts between 5 and 8 years of age are in line with the results of Nardini et al. [38] who found that 6-to-8-year-olds, but not 4-to-5-year-olds could find a goal location from a new starting point they had not visited before. In contrast, Negen and colleagues [39] observed allocentric processing already in 4 to 4-and-a-half-year-olds in a more enclosed environment. Moreover, Bullens, Igloi, Berthoz, Postma, and Rondi-Reig [56] reported successful allocentric processing already in 5-year-old children. However, in their virtual navigation study, updating based on self-movement in combination with viewpoint matching, i.e., sequential egocentric processing, could also have led to successful performance, at least in half of the trials. Our finding thus substantiates the view that the use of allocentric navigation based on proximal landmarks depends on the environment, but only starts between 5 and 8 years of age based on proximal landmarks in an open environment. Allocentric navigation based on proximal landmarks thus seems more difficult as compared to reorientation based on geometry or geometry and landmarks, which can already be elicited in children younger than 5 years of age (e.g., [21,29]). Moreover, it extends these results by showing that allocentric processing based on proximal landmarks becomes more accurate over time at least until 10 years of age. This finding is in line with other studies showing increased precision in distance estimation from a landmark with age in different types of paradigms [16,57]. Our study clearly shows that allocentric processing based on proximal landmarks becomes increasingly accurate with age from 5 until 10 years of age, both with regards to the awareness of being rotated (categorical), as well as in representational precision (metrical). 

In addition to age, sex was also found to be related to performance. The analyses on the rotations showed that, whereas boys and girls did not differ when egocentric navigation strategies could be used, boys outperformed girls in metrical allocentric processing and showed a smaller difference between categorical egocentric and allocentric processing than girls. This is in line with findings by van Dun and colleagues [18] who showed that boys between 9 and 11 years of age outperform girls in a virtual spatial navigation task (see also [17]), and research in adults showing superior performance in allocentric tasks in males in combination with equal performance on egocentric tasks [3,4,5,58]. However, diverging results are also found in children, including no sex differences in performance [15,16], better navigational skills in girls [52,59], and the use of different navigational strategies between girls and boys [60,61,62]. These latter studies assessed a wide variety of navigational behaviors, but not necessarily accurate allocentric processing based on landmarks in a more difficult task. Our results suggest that in sufficiently difficult tasks, like the ones used in adults [3,4,5,58] as well as our paradigm, men and boys may outperform women and girls. This may be due to the larger-scale environments in which adult studies often take place. Better performance of boys can be in these larger environments explained by the hunter-gatherer hypothesis which states that men are better at navigating large-scale environments whereas women perform better in small-scale environments [63]. Another reason for the differences in performance may be the time pressure participants faced. Although speed was not emphasized, there was a time limit in the encoding and returning phase. Previous research has suggested that time pressure affects navigation performance to a larger extent in women compared to men [64,65]. Future research should investigate whether the size of the environment and the presence of time pressure affect sex differences and test whether this is already the case in early development as well. 

Whereas, in general, boys outperformed girls, the preferred cue type did not differ between boys and girls. Both sexes performed better when directional information, i.e., shadows (either alone or in combination with positional information), was available, as compared to when only positional information, i.e., color, was available. The size of this effect was smaller than the effects of Age and Sex, but still, a medium effect was found. In adult studies, a preference for directional cues is shown for men, while a preference for positional cues is often found for women [4,44,45]. In these studies, the locations of positional cues and directional cues generally differ with positional cues being closer to the navigator as compared to the directional cues, while in our study they are in the same location. This suggests that women may prefer cues that are close by as opposed to far away, not necessarily meaning that they prefer positional information over directional information. In line with this, research has also shown that men outperform women in visual processing when information is presented further away, whereas women perform better when visual information is presented nearby [66]. However, biological and social theories also have additional explanations for existing sex differences (see [67] for a review).

The results of the MANCOVAs revealed that, in addition to age (and sex), both spatial working memory and the verbal production of left and right were associated with some forms of navigational performance. Visuo-spatial working memory is related to egocentric metrical and categorical performance. This may be due to the fact that the working memory task used here measures the location of an object with respect to the observer in locations marked by boundaries, and hence only requires egocentric, categorical processing. However, previous studies in adults found impeded egocentric and allocentric navigational performance during spatial interference tasks [10,12,13,14], which suggests that spatial working memory is involved to some extent in allocentric navigation as well, at least at an older age.

Verbal working memory did not relate to performance. In previous studies in adults, performance in navigation tasks was impaired during a verbal working memory interference task, at least in poor navigators [10,11,13]. This discrepancy could indicate a difference in strategy use between children and adults. However, in the current study, we did not assess navigational performance during the execution of a verbal working memory or verbal shadowing task. Therefore, it could also be that a basic level of verbal working memory is employed during navigation, which caused impaired performance in a dual-task paradigm in adults, but did not place a burden beyond the limits of the children’s working memory in our paradigm. Future research should investigate which of these explanations holds by using a dual-task paradigm in children.

In children, previous studies have found effects of left-right knowledge on navigation skills [20], and this effect of correct left-right production (understanding of the concept, not linguistic production) was replicated in our study. The effect was mainly driven by the youngest children since ceiling performance on the left-right task was already reached in the 7- and 8-year-olds. The use of left and right was only associated with categorical performance and not metrical performance. This effect was found for egocentric processing and after 180-degree rotation. Whereas egocentric navigation seems to be possible without coding for left and right as well, as can be inferred from the relatively successful egocentric results in the youngest age group where left-right knowledge was often lacking, verbal coding of left and right seems to facilitate navigation. The predictive value for only categorical but not metrical performance was expected since left and right cannot be used to code for the exact goal location. Although coding of left and right was not expected to predict allocentric performance, it can also easily help to find the goal location after a 180-degree rotation, since one would just need to recognize that a 180-degree rotation angle has taken place and then walk in the direction opposite the remembered direction. In the 90-degree rotation, this is not efficient, since it is more difficult to recognize a rotation as being exactly 90 degrees as compared to exactly 180 degrees. Furthermore, left and right cannot just be switched after the 90-degree rotation.

This study was performed in a desktop virtual environment in which the participants did not physically move. Although one may argue that this kind of navigation does not resemble navigation in the outside world, virtual environments are used in a substantial number of studies in both adults (e.g., [41,68,69]) and children (e.g., [56,70,71]). The use of virtual environments has many advantages, one of which is the ability to control and manipulate variables in the environment [72]. One of the disadvantages of using a virtual environment is the lack of proprioceptive input. Therefore, path integration may not occur as naturally as in real environments. Moreover, performance would potentially depend on the participants’ computer skills. However, previous research directly comparing navigation skills in virtual and real environments found that both adults and children treat virtual environments similarly to real environments [16,73,74,75]. Whereas some of these studies used immersive virtual environments, others, including ours, used non-immersive environments (i.e., desktop computers/laptop screens). The limited body of research available shows that there seems to be no difference in spatial learning within these two types of virtual environments [76]. However, additional research, also in children, is needed to enhance our knowledge of different types of environments. In the current study, the analyses of keyboard use and reported gaming experience do not explain children’s performance on the task. However, future research that assesses gaming experience more elaborately, for example by including the types of games that are played, is suggested.

## 5. Conclusions

This study is the first to give insight into the development of allocentric navigation skills based on proximal landmarks and the relative contributions of spatial working memory, verbal working memory and left-right knowledge on these skills in boys and girls between 5 and 10 years of age. The results indicate that the mental map of the environment becomes more precise with age. Boys displayed more accurate allocentric navigation skills as compared to girls. Left-right knowledge was related to navigational performance after 0- and 180-degrees rotation, indicating that children who do code for left and right are better able to navigate, at least at a coarse level. Moreover, visuo-spatial working memory was related to egocentric navigation, but not allocentric navigation. In contrast to other studies, verbal working memory was not related to navigational performance. This study shows the importance of including several possible covariates together in one study to examine their relative importance. The results show early advantages in allocentric navigation for boys as compared to girls indicating that it may be useful to train girls on navigation skills from a young age. Future research should examine the potential benefit of such training. Moreover, future research using different navigation tasks could give additional insight into the stability of covariates of navigational performance.

## Figures and Tables

**Table 1:** Means and standard deviations (in brackets) for each age group on the experimental tasks.

	6 Years	8 Years	10 Years
AWMA backwards digit recall	102.28 (18.26)	106.69 (13.02)	100.17 (12.09)
AWMA odd one out	109.87 (15.60)	116.52 (12.87)	111.91 (15.05)
Left-right task	4.95 (3.14)	6.95 (2.53)	7.59 (1.75)

**Table 2:** Repeated measures MANOVA on Rotations.

	*df*	*F*	*p*	*η* ^2^
Rotation	4, 103	49.60	<0.001 **	0.658
Metrical	2, 212	73.89	<0.001 **	0.411
Categorical	2, 212	109.69	<0.001 **	0.509
Age Group	4, 210	17.93	<0.001 **	0.222
Metrical	2, 105	42.20	<0.001 **	0.443
Categorical	2, 105	34.18	<0.001 **	0.392
Sex	2, 105	9.35	<0.001 **	0.151
Metrical	1, 106	12.17	0.001 *	0.103
Categorical	1, 106	2.98	0.087 ^†^	0.027
Rotation *Age Group	8, 206	1.89	0.063 ^†^	0.067
Metrical	4, 103	3.49	0.009 *	0.062
Categorical	4, 103	3.83	0.005 *	0.067
Rotation *Sex	4, 103	2.54	0.045 *	0.090
Metrical	2, 212	4.25	0.016 *	0.039
Categorical	2, 212	4.82	0.009 *	0.044
Rotation *Age Group *Sex	8, 206	0.54	0.829	0.020

† *p* < 0.10, * *p* < 0.05, ** *p* < 0.001.

**Table 3:** MANCOVA analyses on metrical and categorical errors.

	Metrical				Categorical			
	*df*	*F*	*p*	*η* ^2^	*df*	*F*	*p*	*η* ^2^
Sex	3, 85	2.69	0.051 ^†^	0.087	3, 86	0.95	0.420	0.032
Age	3, 85	9.37	<0.001 **	0.248	3, 86	6.48	0.001 *	0.184
Verbal WM	3, 85	0.45	0.721	0.015	3, 86	1.58	0.199	0.052
Spatial WM	3, 85	2.18	0.097 ^†^	0.071	3, 86	2.98	0.036 *	0.094
Left/right	3, 85	1.94	0.129	0.064	3, 86	4.52	0.005 *	0.136
Keyboard use	3, 85	0.39	0.764	0.013	3, 86	0.40	0.752	0.014
Game experience	3, 85	0.08	0.969	0.003	3, 86	0.44	0.726	0.015

† *p* < 0.10, * *p* < 0.05, ** *p* < 0.001.

**Table 4:** Repeated measures MANOVA on Cue Type.

	*df*	*F*	*p*	*η* ^2^
Cue Type	4, 103	5.65	<0.001 **	0.180
Metrical	2, 212	11.99	<0.001 **	0.102
Categorical	2, 212	11.23	<0.001 **	0.096
Age Group	4, 212	15.43	<0.001 **	0.226
Metrical	2, 106	43.34	<0.001 **	0.450
Categorical	2, 106	30.90	<0.001 **	0.368
Sex	2, 105	11.99	<0.001 **	0.186
Metrical	1, 106	16.48	<0.001 **	0.135
Categorical	1, 106	4.17	0.044 *	0.038
Cue Type *Age Group	8, 206	1.16	0.322	0.043
Cue Type *Sex	4, 103	0.53	0.712	0.020
Cue Type *Age Group *Sex	8, 206	0.38	0.926	0.015

* *p* < 0.05, ** *p* < 0.001.

## Data Availability

The data presented in this study are available on request from the corresponding author. The data are not publicly available because no written consent for public data sharing was obtained from the parents of the participating children.
